# The first discovery of Tc1 transposons in yeast

**DOI:** 10.3389/fmicb.2023.1141495

**Published:** 2023-02-16

**Authors:** Jia Chang, Guangyou Duan, Wenjing Li, Tung On Yau, Chang Liu, Jianlin Cui, Huaijun Xue, Wenjun Bu, Yanping Hu, Shan Gao

**Affiliations:** ^1^College of Life Sciences, Nankai University, Tianjin, China; ^2^School of Life Sciences, Qilu Normal University, Jinan, Shandong, China; ^3^Qinghai Provincial Key Laboratory of Qinghai-Tibet Plateau Biological Resources, Northwest Institute of Plateau Biology, Chinese Academy of Sciences, Xining, Qinghai, China; ^4^Key Laboratory of Adaptation and Evolution of Plateau Biota, Northwest Institute of Plateau Biology, Chinese Academy of Sciences, Xining, Qinghai, China; ^5^Department of Rural Land Use, Scotland’s Rural College, Aberdeen, United Kingdom; ^6^School of Medicine, Nankai University, Tianjin, China

**Keywords:** methylotrophic yeast, transposase, long terminal repeat, terminal inverted repeat, IS630

## Abstract

**Background:**

Identification of transposons without close homologs is still a difficult task. IS630/Tc1/mariner transposons, classified into a superfamily, are probably the most widespread DNA transposons in nature. Tc1/mariner transposons have been discovered in animals, plants, and filamentous fungi, however, not in yeast.

**Results:**

In the present study, we report the discovery of two intact Tc1 transposons in yeast and filamentous fungi, respectively. The first one, named Tc1-OP1 (DD40E), represents Tc1 transposons in *Ogataea parapolymorpha*. The second one, named Tc1-MP1 (DD34E), represents Tc1 transposons in the *Rhizopodaceae* and *Mucoraceae* families. As a homolog of Tc1-OP1 and Tc1-MP1, IS630-AB1 (DD34E) was discovered as an IS630 transposon in *Acinetobacter* spp.

**Conclusion:**

Tc1-OP1 is not only the first reported Tc1 transposon in yeast, but also the first reported nonclassical Tc1 transposon. Tc1-OP1 is the largest of IS630/Tc1/mariner transposons reported to date and significantly different from others. Notably, Tc1-OP1 encodes a serine-rich domain and a transposase, extending the current knowledge of Tc1 transposons. The phylogenetic relationships of Tc1-OP1, Tc1-MP1 and IS630-AB1 indicated that these transposons had evolved from a common ancestor. Tc1-OP1, Tc1-MP1 and IS630-AB1 can be used as reference sequences to facilitate the identification of IS630/Tc1/mariner transposons. More Tc1/mariner transposons will be identified in yeast, following our discovery.

## Introduction

Transposable elements (TEs), also called transposons, represent a substantial fraction of eukaryotic genomes (Marini et al., 2010) and can influence many aspects of DNA function that range from the evolution of genetic information to duplication, stability, and gene expression. The identification and characterization of transposons is a major genomics subject for basic research or engineering applications. Basically, transposons are grouped into two classes: class I transposons, also called retrotransposons and class II transposons, also called DNA transposons. Class II can be divided into subclass 1, 2, and 3, and subclass 1 has two orders - terminal inverted repeat (TIR) and Crypton. The order TIR is further divided into superfamilies. Tc1/mariner, as one of TIR superfamilies, represents probably the most widespread DNA transposons in nature and includes at least three (Tc1, mariner and pogo) families (Plasterk et al., 1999). Tc1 and mariner, as two families, are defined by the featured domains of their transposases, which contain the catalytic pockets responsible for cleaving DNA strands. The featured domains of Tc1 and mariner transposases have active site motifs that consist of three acidic amino-acid (aa) residues DDE and DDD, respectively. The first Tc1 transposon was discovered in *Caenorhabditis elegans* in 1983 (Emmons et al., 1983), while the first mariner transposon was discovered in *Drosophila mauriliana* (Jacobson et al., 1986). Subsequently, Tc1/mariner transposons were discovered in plants and filamentous fungi. The famous sleeping beauty (SB) system was developed as a gene transfer tool using the SB transposon, which is a synthetic transposon that had been constructed based on the sequences of inactive Tc1 transposons discovered in fish genomes (Ivics et al., 1997). However, many of the reported transposons were discovered without their nucleotide (nt) sequences. For instance, the nt sequence of the first discovered Tc1 transposon in *C. elegans* had not been provided with the published article (Emmons et al., 1983). More nt sequences are required as references to discover or identify transposons in various species for further research.

With the wide application of PacBio and Nanopore DNA-seq (Xu et al., 2019), a great number of complete or even full-length genomes have been increasingly submitted to the public databases (Chang et al., 2022). These genomes are facilitating the discovery or identification of transposons in various species. Particularly, the nt sequences of the reported TIRs, long terminal repeats (LTRs), transposases, or other components are being collected into repeat-sequence databases [e.g., Repbase (Bao et al., 2015)], which are used as references to discover or identify new transposons by sequence homology search. As many of transposons that have been inactivated during evolution and altered beyond recognition over a long period of time, identification of transposons without close homologs is still a difficult task. Due to the above reason, the identification and characterization of transposons in fungi lag behind that in plants and animals. Retrotransposons have been well studied in both filamentous fungi and yeast (Neuvéglise et al., 2002), while DNA transposons have been intensively studied in filamentous fungi (Daboussi and Capy, 2003), but scarcely in yeast, probably because it has been well accepted that *Saccharomyces cerevisiae* lacks DNA transposons (Weil and Kunze, 2000). Among the identified DNA transposons in filamentous fungi, impala is a representative of the Tc1 transposons, whose autonomous members have up to 20% of divergence at the DNA level (Hua-van et al., 1998). However, Tc1/mariner transposons have not been discovered in yeast.

In our previous study (Chang et al., 2022), we assembled the full-length genome of *Ogataea polymorpha* HU-11/CBS4732 using high-depth PacBio data. With this high-quality genome, the relationship between three basic *O. polymorpha* strains (CBS4732, NCYC495, and DL-1; Massoud et al., 2003) was determined and protein-coding, rRNA genes, and retrotransposons were used as signatures to discriminate *O. parapolymorpha* DL-1 from *O. polymorpha* CBS4732 and NCYC495. Unexpectedly, we discovered an intact Tc1 transposon in *O. parapolymorpha* DL-1, which was named as Tc1-OP1 (OP stands for *O. parapolymorpha*). As Tc1-OP1 is present in DL-1, but absent in CBS4732 and NCYC495, it can be used as a genetic marker to clearly discriminate DL-1 from CBS4732 and NCYC495. In the present study, we characterized Tc1-OP1 with more comprehensive and accurate information: (1) to help researchers to identify Tc1 transposons using Tc1-OP1 as reference; (2) to provide a nonclassical model of Tc1 transposons, which merit further investigation; and (3) to provide a basis for the development of new gene transfer systems, particularly for *Ogataea* spp., as many of them have been genetically engineered for industrial application (Massoud et al., 2003).

## Results and discussion

### Discovery of Tc1-OP1 in yeast

**Figure 1:**
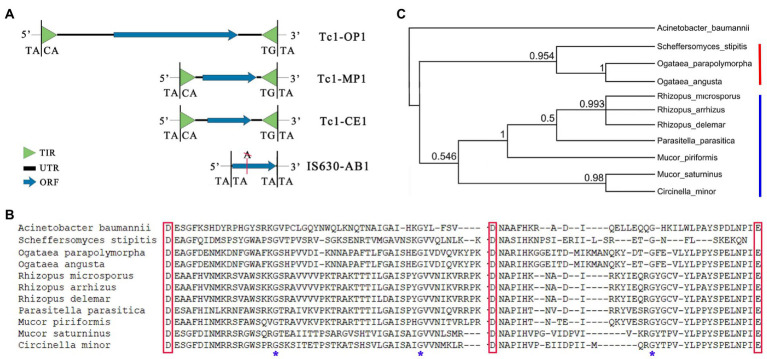
Tc1-OP1, Tc1-MP1, Tc1-CE1, and IS630-AB1. **(A)** Tc1-OP1, Tc1-MP1, and Tc1-CE1 are Tc1 transposons discovered in *Ogataea polymorpha* DL-1, *M. piriformis*, and *C. elegans*, respectively. IS630-AB1 is an IS630 transposon discovered in *Acinetobacter baumannii* CAM180-1. The nt sequences of them have lengths of 5,659, 1,688, 1,570, and 884 bp, respectively. Tc1-OP1, Tc1-MP1, and IS630-AB1 are reported in the present study, while Tc1-CE1 was reported in a previous study. Although IS630-AB1 does not contain TIRs, it contains the excision sites “TA|TA” and “TA|TA” at its 5′ and 3′ ends, respectively. TIR: terminal inverted repeat; UTR: untranslated region; ORF: open reading frame. **(B)** Multiple alignment of the DDE domains of 10 Tc1 transposases in fungi exhibited a higher degree of conservation in their aa sequences (Supplementary File S1). A notable feature of these DDE domains is that they contain three highly conserved glycine residues (Indicated by asterisks). The DDE domains of *A. baumannii* 625,974 and IS630-AB1 have identical aa sequences. These aa sequences were used for the next phylogenetic analysis. **(C)** The aa sequences of the 10 DDE domains from fungi were used for the phylogenetic analysis, with the DDE domain of IS630-AB1 as an outgroup. The tree topology was recovered by Bayesian inference (BI) analyses. The posterior probabilities of all nodes were showed in decimal format. Clade I (indicated by red line) includes the DDE domains from *Ogataea parapolymorpha* (Tc1-OP1), *O. angusta* (GenBank: KAG7805855) and *Scheffersomyces stipitis* (GenBank: XP_001384294), while Clade II (indicated by blue line) includes the DDE domains from *Rhizopus microsporus* (GenBank: KAG1255894), *R. arrhizus* (GenBank: KAG1532800), *R. delemar* (GenBank: KAG1490818), *Parasitella parasitica* (GenBank: CEP14362.1), *Mucor piriformis* (Tc1-MP1), *M. saturninus* (GenBank: KAG2191411), and *Circinella minor* (GenBank: KAG2210425).

Genomic comparison revealed that a 3,468-bp open reading frame (ORF) is present in the complete genome of *O. parapolymorpha* DL-1 (GenBank: CP080317-21), but absent in *O. polymorpha* CBS4732 and NCYC495 (Chang et al., 2022). This 3,468-bp ORF was incorrectly annotated as a 3,054-bp ORF in the GenBank database. Using InterProScan tool (Methods and materials), the 3,468-bp ORF was identified as a putative transposase containing a DDE domain by its homolog (UniprotKB: A0A009PQK7) from *Acinetobacter baumannii* 625,974. A 5,659-bp genomic region (CP080317: 544314–549,972) containing the 3,468-bp ORF was discovered as a Tc1 transposon and named as Tc1-OP1 (OP stands for *O. parapolymorpha*), based on the following evidence: (1) Tc1-OP1 has the identical 169-bp TIRs at 5′ and 3′ ends; (2) the 5′ and 3’ TIRs of Tc1-OP1 are flanked by the excision sites “TACA|” and “|TGTA,” respectively; and (3) the 3,468-bp ORF encodes a Tc1 transposase. The ORF in Tc1-OP1, if beginning with the start-codon “ATG,” has a length of 3,468 bp, and if beginning with the codon “TGG,” has a length of 3,516 bp, extending upstream by 48 bp (16 aa). Tc1-OP1 is the largest of Tc1/mariner transposons reported to date and it is even larger than OSMAR1 from *Oryza sativa* L. (cultivated rice; Feschotte et al., 2003), which is the largest mariner transposon reported to date. The 5,274-bp reference sequence of OSMAR1 (Supplementary File S1) includes a 33-bp 5’ TIR, a 1,806-bp 5′ untranslated region (UTR), a 1,824-bp ORF (607 aa), a 1,579-bp 3’ UTR, and a 33-bp 3’ TIR, while the 5,659-bp reference sequence of Tc1-OP1 includes a 169-bp 5’ TIR, a 245-bp 5’ UTR, a 3,468-bp ORF (1,155 aa), a 1,608-bp 3’ UTR, and a 169-bp 3’ TIR (Figure 1A). In total, two copies of Tc1-OP1 were located in the chromosome 1 (CP080317: 544314–549,972) and 3 (CP080318: 300099–305,754), which were designated as copy 1 and 2 (Supplementary File S1), respectively. In addition, very few solo TIRs and no partial sequences of Tc1-OP1 were detected in the genome of *O. parapolymorpha* DL-1. Compared to copy 1, copy 2 had 46 single nucleotide polymorphisms (SNPs) and a deletion of 3 bp in its sequence, which resulted in the deletion of only a methionine (M) residue in the encoded protein. The complete genome of DL-1 (GenBank: CP080317-21) was assembled using Nanopore DNA-seq data, excluding the possibility of Tc1-OP1 misassembly. Two copies of Tc1-OP1 were also detected in another complete genome of DL-1 (RefSeq: NC_027860–66), confirming the above results.

By blastp, the homologs of Tc1-OP1 in filamentous fungi were identified as Tc1 transposons. The homolog from *Mucor piriformis* was designated as the reference of these Tc1 transposons and named as Tc1-MP1 (MP stands for *M. piriformis*) in the present study. The nt sequence of Tc1-MP1 (OW971871: 2795447–2,793,760) was determined using the complete genome of *M. piriformis* (GenBank: OW971867-12). This 1,688-bp reference sequence (Supplementary File S1) includes a 141-bp 5’ TIR, a 60-bp 5’ UTR, a 1,305-bp ORF (434 aa), a 41-bp 3’ UTR, and a 141-bp 3’ TIR (Figure 1A). The DDE domains of Tc1-OP1 and Tc1-MP1 share an aa identity of 41.83% (64/153) and a positive-substitution percentage of 54.9% (84/153). As Tc1-OP1 and Tc1-MP1 contained significantly different TIRs and ORFs, they represent two distinct groups of Tc1 transposons in fungi. To make comparison between Tc1-OP1, Tc1-MP1 and Tc1 transposons from *C. elegans*, a reference was required to be designated and named as Tc1-CE1 (CE stands for *C. elegans*) in the present study. However, the nt sequence of the first discovered Tc1 transposon in *C. elegans* had not been provided with the published article (Emmons et al., 1983). By searching the public databases, we obtained the aa sequence of Tc1-CE1 transposase (PIR: F89402) predicted from the expressed sequence tags (ESTs) of *C. elegans*. Using tblastn, this aa sequence was aligned to the complete genome of *C. elegans* (RefSeq: NC_003279–84), obtaining at least 30 copies of Tc1-CE1. All these 30 copies contain the same ORF which had a 41-bp insertion, resulting in a frame-shift mutation. After removal of the 41-bp insertion, the nt sequence corresponding to the predicted transposase (PIR: F89402) and its flanking sequences obtained from the complete genome (NC_003283: 17805799–17,807,409) were used to recover the nt sequence of Tc1-CE1. This 1,570-bp reference sequence (Supplementary File S1) includes a 26-bp 5’ TIR, a 246-bp 5’ UTR, a 1,032-bp ORF (343 aa), a 240-bp 3’ UTR, and a 26-bp 3’ TIR (Figure 1A). According to previous studies, with a few exceptions [e.g., DD37D, DD37E, DD38E, and DD39D (Shao and Zhijian, 2001)], all Tc1 transposases identified in fungi, invertebrates, and vertebrates contain a DD34E motif, while most mariner transposases identified in flatworm, insects, and vertebrates contain a DD34D motif. A simple comparison of Tc1-OP1 (DD40E), Tc1-MP1 (DD34E), Tc1-CE1 (DD34E) and the transposon containing the DDE domain from *A. baumannii* 625,974 (DD34E) showed their lengths of 5,659, 1,688, 1,570, and < 1,500 bp, their encoded-protein lengths of 1,155, 434, 343, and < 340 aa, and their DDE-domain lengths of 206, 143, 126 and 116 aa, respectively (Figure 1B). This significant difference in lengths of the DDE domains merits further investigation. Notably, the single protein encoded by Tc1-OP1 is 2.66 (1,155/434) times larger than that encoded by Tc1-MP1. Thus, the single protein encoded by Tc1-OP1 was named as the Tc1-OP1 protein, and the region (734–1,155 aa) of the Tc1-OP1 protein was named as the Tc1-OP1 transposase (Further analyzed below). As Tc1-OP1 (DD40E) is significantly different from all other Tc1/mariner transposons reported to date, it is the first reported nonclassical Tc1 transposon.

### Discovery of IS630-AB1 in bacteria

To make comparison of Tc1-OP1, Tc1-MP1, and their homologs in bacteria, the genome of *A. baumannii* 625,974 was required, however, it was not available. We had to use the genomes of its closest species to recover the nt sequences of transposons in *Acinetobacter* spp. Using tblastn, the aa sequence of the DDE domain from *A. baumannii* 625,974 was aligned to the genomes of *Acinetobacter* spp., resulting in the acquisition of a large number of genomic regions. These genomic regions encode the homologs of Tc1-OP1 and Tc1-MP1 in *Acinetobacter* spp., which were identified as transposons belonging to the IS630 family. The first IS630 transposon was discovered in the *Shigella sonnei* genome (Matsutani et al., 1987). Subsequently, with more and more identified IS630 family members showing similarities to Tc1 transposons, IS630, Tc1 and mariner families have been reclassified as the IS630/Tc1/mariner superfamily. IS630 is a family of insertion sequence (IS) elements in bacteria, which usually occur as parts of composite transposons. An IS630 composite transposon usually contain two copies of IS630 transposons (i.e., IS630L and IS630R). However, each of the above *Acinetobacter* genomes does not contain IS630 composite transposons. Notably, the genomes of *A. seifertii*, *A. baumannii*, *A. pittii*, *A. ursingii*, and *A. lwoffii* contain only single IS630 transposons, which contains only one ORF. while the genomes of *A. seifertii*, *A. baumannii*, and *A. nosocomialis* contain only partial sequences of single IS630 transposons.

**Figure 2:**
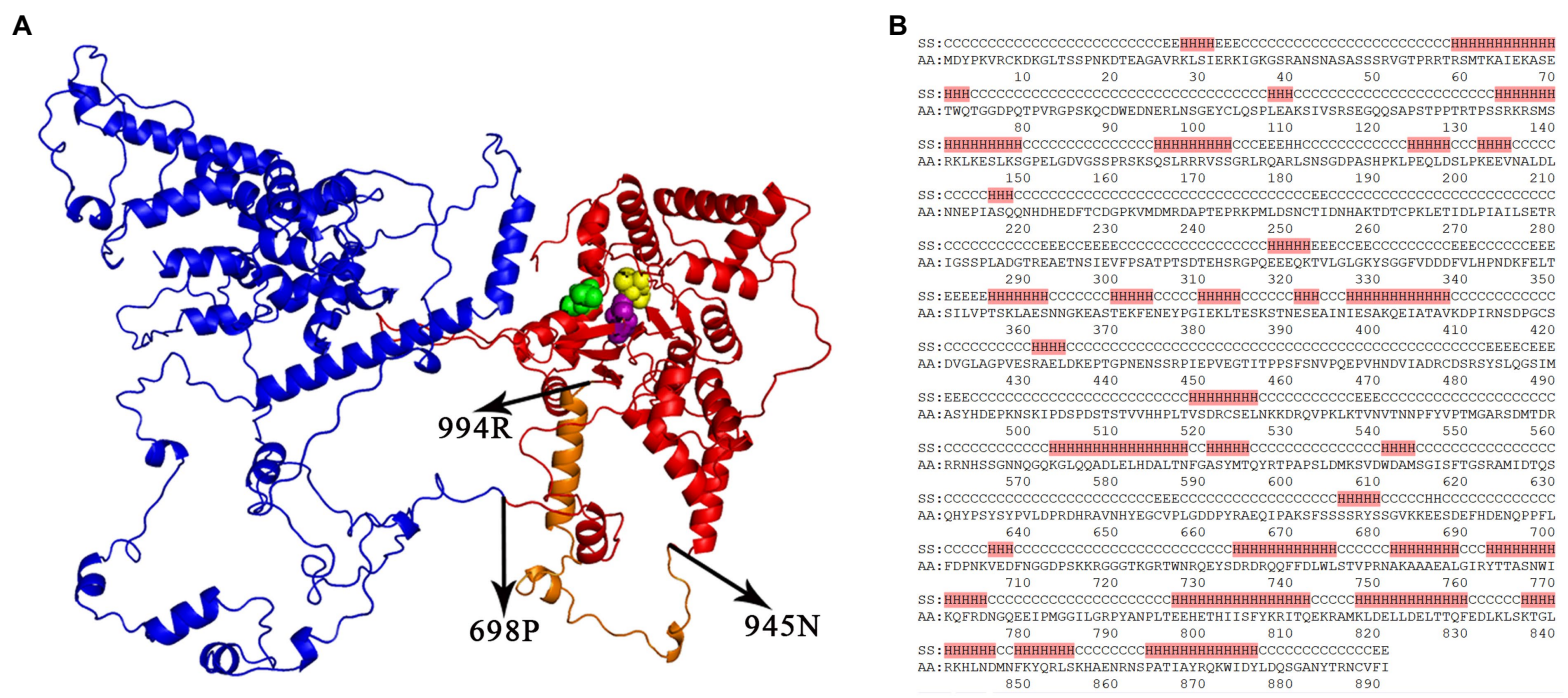
The 1155-aa protein encoded by Tc1-OP1. An unknown region (named domainX) spanning 1–681 aa and the Tc1-OP1 transposase spanning 734–1,155 aa were separated by a junction region (682–733 aa) of the 1,155-aa protein encoded by Tc1-OP1, named the Tc1-OP1 protein. **(A)** The 3D structures of domainX (Blue) and the Tc1-OP1 transposase (Red) were predicted. The 892–1,097 aa is the DDE domain with three catalytic aa residues 892D (Purple), 1056D (Yellow) and 1097E (Green). The region (945–994 aa) is a 50-aa insert (Orange) in the Tc1-OP1 transposase, which is absent in the Tc1-MP1, Tc1-CE1, and IS630-AB1 transposases. 3D structures of the Tc1-OP1, Tc1-MP1, and Tc1-CE1 transposases have been provided in Supplementary File S2. Tc1-OP1 protein (139–1,155 aa) predicted by trRosettaX had the best template modeling (TM) score of 0.334. **(B)** The secondary structures were predicted for 1–891 aa of the Tc1-OP1 protein. A possible N-terminal DNA-binding domain (734–891 aa) contains two HTH motifs (734–775 and 798–855 aa). H, alpha helix; E, beta sheet; C, coil; SS, predicted secondary structure; AA, amino acid residue.

The homolog from *A. baumannii* CAM180-1 was designated as the reference of the IS630 transposons in *Acinetobacter* spp. and named as IS630-AB1 (AB stands for *A. baumannii*) in the present study. To recover the nt sequence of IS630-AB1, a 2,161-bp region (CP044356: 172775–174,935) was acquired from the complete genome of *A. baumannii* CAM180-1 (GenBank: CP044356.1), containing the upstream and downstream sequences of IS630-AB1. The 2,161-bp region included a 642-bp ORF1 (213 aa), a 347-bp noncoding region, a 291-bp ORF2a (97 aa), a 14-bp noncoding region, a 555-bp ORF2b (184 aa), a 24 bp noncoding region and a 288-bp ORF3 (95 aa). ORF1 and ORF3 were identified as a DedA family protein (UniprotKB: F0KJE3) and a DoxX family protein (UniprotKB: F0KJE4), respectively, while ORF2a and ORF2b were identified as two parts of the IS630-AB1 transposase, as two insertions of adenine (A) had resulted in the break of ORF2 into ORF2a and ORF2b (Figure 2A). This indicated that IS630-AB1 had been inactivated through evolution, so its TIRs may have altered beyond recognition, which was confirmed by the results that no IS630 TIRs were detected in the genomes of *Acinetobacter* spp. using RepeatMasker (Methods and materials). Then, we had to locate the boundaries of IS630-AB1 in the 2,161-bp region without knowing its TIRs. Finally, the boundaries of IS630-AB1 were determined on the basis of sequence comparisons between two major groups of homologs of the 2,161-bp region from the NCBI GenBank and RefSeq databases. All these homologs were only identified in *Acinetobacter* spp. The first major group of homologs have almost 100% nt identities with the 2,161-bp region, while the second major group have a 886-bp deletion (CP044356: 173741–174,626), corresponding to the lost IS630-AB1. Thus, the 886-bp segment was used to recover the nt sequence of IS630-AB1. After removal of the two A residues, the 884-bp nt sequence of IS630-AB1 (Supplementary File S1) was determined, including a 23-bp 5’ UTR, a 858-bp ORF and a 3-bp 3’ UTR (Figure 1A). Although the TIRs of IS630-AB1 are still unknown, the boundaries of IS630-AB1 were confirmed by the excision sites “TA|TA” and “TA|TA” (Urasaki et al., 2002) at its 5′ and 3′ ends, respectively (Figure 1A). The length (884 bp) of IS630-AB1 is close to the average length (1,100 bp) of IS630 members, which have the min and max lengths of 950 and 1,250 bp, respectively. Furthermore, the excision sites of “TA|TA” of IS630-AB1 were confirmed to be present in all genomes containing the first major group of homologs, by comparing the two major groups of homologs.

### Homologs of Tc1-OP1 and Tc1-MP1 in fungi

Using blastp, the Tc1-OP1 protein (1,155 aa) and the Tc1-MP1 transposase (434 aa) were used to search for homologs in the NCBI NR database. The homologs of the Tc1-OP1 protein were only identified in yeast (i.e., *O. parapolymorpha* and *O. angusta*) with aa identities above 80%. In previous studies, *O. polymorpha* and *parapolymorpha* strains were not discriminated and some of them were named as *O. angusta* strains. As the full-length homologs of the Tc1-OP1 protein were only identified in *parapolymorpha* of the *Ogataea* genus, including *O. parapolymorpha* 73–26 (GenBank: KAG7866721), CBS11895 (GenBank: KAG7863972), and CBS12304 (GenBank: KAG7871386), *etc*, we inferred that all the *O. angusta* strains containing the homologs of the Tc1-OP1 protein with aa identities above 80% are *O. parapolymorpha* strains. For instance, *O. angusta* 60–394 (GenBank: KAG7834541), 61–244 (GenBank: XP_043058079), and CBS2575 (GenBank: KAG7805855) containing the homologs of the Tc1-OP1 protein with aa identities of 87% (1,014/1162), 87% (1,014/1162), and 87% (960/1102), respectively, are *O. parapolymorpha* strains. More high-quality genomes are required to be used to confirm that Tc1-OP1 can be used as a genetic marker to discriminate *parapolymorpha* from *polymorpha* or other closely related species. In addition to the *Ogataea* genus, homologs of the Tc1-OP1 protein were also identified in some other yeast, such as *Scheffersomyces stipitis* (Figure 1B). However, it does not merit further investigation, as its aa sequence is incomplete and has not yet been subject to final NCBI review. The homologs of the Tc1-MP1 transposase were only identified in filamentous fungi, including *Rhizopus microsporus*, *R. arrhizus*, *R. delemar*, *Parasitella parasitica*, *Mucor saturninus*, *Circinella minor*, etc. These homologs shared aa identities above 60% with the Tc1-MP1 transposase.

After removal of sequence redundancy, transposases encoded by Tc1 transposons from 10 fungi species were selected for phylogenetic analysis. Multiple sequence alignment of their DDE domains (Figure 1B) exhibited a higher degree of conservation in their aa sequences, particularly around the aspartate (D), D and glutamate (E). A notable feature of these DDE domains is that they contain three highly conserved glycine residues (e.g., 910G, 935G and 1083G in Tc1-OP1). The aa sequences of the 10 DDE domains in fungi were used for the phylogenetic analysis (Methods and materials), with the DDE domain of IS630-AB1 as an outgroup. The tree topology (Figure 1C) was recovered by Bayesian inference (BI) analyses. All the 10 DDE domains from fungi were grouped into two clades — clade I (i.e., the Tc1-OP1 clade) including the DDE domains from yeast, and clade II (i.e., the Tc1-MP1 clade) including the DDE domains from the *Rhizopodaceae* and *Mucoraceae* families. These results confirmed that Tc1-OP1 and Tc1-MP1 represent Tc1 transposons in yeast and filamentous fungi, respectively. Due to the limitation of the data availability, clade I almost does not include the DDE domains from species outside the *Ogataea* genera. Although the DDE domain from *Scheffersomyces stipitis* (RefSeq: XP_001384294.2) was also classified into clade I with a high posterior probability, it does not merit further investigation, as its aa sequence is incomplete (Figure 1B) and has not yet been subject to final NCBI review. In Clade II, the DDE domains from *R. microsporus*, *R. arrhizus*, *R. delemar* were grouped into a branch, however, those from *M. saturninus* and *M. piriformis* were not grouped into a branch. Unexpectedly, the DDE domains from *M. saturninus* and *C. minor* were grouped into a branch, while the DDE domain from *M. piriformis* was closer to that from *P. parasitica*, than that from *M. saturninus*. The identification of *Parasitella*, *Mucor*, and *Circinella* strains is often confused, as they all belong to the *Mucoraceae* family and share common features. Thus, Tc1-MP1 helps to improve the identification of strains in the *Rhizopodaceae* and *Mucoraceae* families.

### Tc1-OP1 is a nonclassical DNA transposon

According to the classical model, a Tc1 transposon encodes a transposase. An intact Tc1/mariner transposase contains at least a N-terminal DNA-binding domain and a DDE/DDD domain. The DDE/DDD domains are highly conserved in their sequences (Figure 1B) and structures, so they are used to identify Tc1/mariner transposons. Compared to the well-studied DDE/DDD domains, the components of the N-terminal DNA-binding domains are controversial and their diversity is still unclear. According to previous studies (Plasterk et al., 1999): some bacterial and Pogo transposases contain a ‘solo’ helix–turn–helix (HTH) motif in their N-terminal DNA-binding domains; mariner transposases contain two HTH motifs; and Tc1 transposases contain a HTH motif to form a dimer and another HTH motif that is embedded in a homeo-like DNA-binding domain. However, the knowledge mentioned above has been obtained based on sequence alignments and secondary-structure predictions using aa sequences from only several species. Therefore, the current knowledge of the N-terminal DNA-binding domains is still very limited, but is being expanded by many new results. For instance, a centromere-binding protein B (CENPB) domain was discovered in eight mariner transposons (named as TremA-H) of the *Paracoccidioides* species complex in a recent study (Marini et al., 2010). As nt or aa sequences of fungi Tc1 transposases are scarcely available in public databases, no information associated with the N-terminal DNA-binding domains in Tc1-OP1 or Tc1-MP1 was acquired by homology search against the NCBI NR, Uniprot databases or analysis of protein domains from Pfam and InterPro databases. Multiple sequence alignment of Tc1-OP1, Tc1-MP1, and IS630-AB1 showed that the first homologous region (Figure 1B) is the DDE domain, which spans 892–1,097 aa of the Tc1-OP1 protein, 235–377 aa of the Tc1-MP1 transposase, and 135–250 aa of the IS630-AB1 transposase. This homologous region is significantly different from the DDE domain of the Tc1-CE1 transposase (157–282 aa). Then, the second homologous region was detected to span the 717–891 aa of the Tc1-OP1 protein, and 62–234 aa of the Tc1-MP1 transposase, however, it has no homologs in IS630-AB1, Tc1-CE1 or TremA-H (Described above). This homologous region may include the N-terminal DNA-binding domains of the Tc1-OP1 and Tc1-MP1 transposases.

The secondary and 3D structures of the Tc1-OP1 protein (1,155 aa) were predicted for further analysis. The predicted 3D structure (Methods and materials) exhibited two domains (Figure 2A) corresponding to an unknown region (1–681 aa) and the Tc1-OP1 transposase (734–1,155 aa), respectively. The unknown region is likely to encode a domain, named domainX. The transposase and domainX are separated by a junction region (682–733 aa). The transposase includes the DDE domain (892–1,097 aa) and a N-terminal DNA-binding domain (734–891 aa) containing two HTH motifs (734–775 and 798–855 aa). The two HTH motifs are consistent with six helix regions (Figure 2B) in the predicted secondary structures (Methods and materials). The DDE domain consists of at least a three-beta-sheet structure CVFIDEAGFD (888–897 aa), AFTLFGAIS (923–931 aa), GIVDVQVK (934–941 aa), which forms the core of the enzyme by supporting three catalytic aa residues 892D, 1056D and 1097E (Figure 2A). Particularly, 892D, 1056D and 1097E are close enough in 3D space for the catalytic function, although they are not close in the aa sequence. Multiple alignment of the DDE domains (Supplementary File S1) showed that a 50-aa insert (945–994 aa) between 892D and 1056D in the Tc1-OP1 protein (Figure 2A) is absent in the Tc1-MP1, Tc1-CE1, and IS630-AB1 transposases, mainly accounting for the size difference between the DDE domain of Tc1-OP1 and those of Tc1-MP1. Tc1-CE1, and IS630-AB1. This insert may form a helix region and a coil region with unknown functions.

Then, we excluded the possibility that Tc1-OP1 belongs to composite transposons (Tansirichaiya et al., 2016). A composite transposon consists of two TIRs from two separate transposons moving together as one unit and carrying the DNA between them. Most composite transposons have been identified in bacteria and can carry catabolic genes and antibiotic resistance genes. Tc1-OP1 is not a composite transposon, as no TIRs or their remnants were detected to flank the nt sequence encoding domainX. We also excluded the possibility that Tc1-OP1 belongs to Mutator-like elements (MULEs) (Dupeyron et al., 2019). Although MULEs usually have one or more additional proteins as Tc1-OP1 has domainX, the proteins in a MULE are not encoded in one ORF as those in Tc1-OP1. Due to inadequate template proteins, the predicted 3D structure of domainX (Figure 2) was not as qualified as those of the Tc1-OP1, Tc1-MP1, Tc1-CE1, and IS630-AB1 transposases, so domainX was still unidentified. Using DaliLite and 3D-BLAST (Methods and materials), we did not obtain adequate information to identify domainX, either. Using SignalP (Methods and materials), no signal peptide was detected in domainX. The analysis of aa composition of domainX showed that the percentages of serine (S), proline (P), E, and D residues reached 13.36, 8.37, 7.34, and 7.2%, respectively, indicating that domainX is a serine-rich protein. Then, we calculated the Pearson correlation coefficients (PCCs) between aa composition of domainX and those of 5,322 proteins annotated in the genome of *O. polymorpha* DL-1 (RefSeq: NC_027860–66). The highest PCC of 0.95 was achieved between domainX and another serine-rich protein (UniprotKB: W1QE49). Serine-rich proteins can be classified into groups, typically including serine/arginine rich (SR-rich) (Isshiki et al., 2006), serine/alanine/proline rich (SAP-rich) (Baida et al., 2006), proline/serine rich (PS-rich) (Luo et al., 2022) and serine/proline rich (SP-rich) proteins (Sahebi et al., 2015). With high percentages of serine and proline, domainX is more likely to be a SP-rich protein rather than a SR-, SAP-, or PS-rich protein. According to a previous study (Sahebi et al., 2015), a SP-rich protein might catalyze the localized depositing associated with silica at the attempted site regarding fungal penetration. On the other hand, as domainX contains a high-percentage of aa residues in coil secondary structures (Figure 2A), it is very likely to function in interactions with other proteins or RNAs. However, the identity of domainX is still undetermined.

## Conclusion

Tc1/mariner transposons have been discovered in animals, plants and filamentous fungi, but not in yeast. In the present study, we report the discovery of two intact Tc1 transposons in yeast and filamentous fungi, respectively. The first one, named Tc1-OP1, represents Tc1 transposons in *Ogataea parapolymorpha*. The second one, named Tc1-MP1, represents Tc1 transposons in the *Rhizopodaceae* and *Mucoraceae* families. Tc1-OP1 and Tc1-MP1 have homologs in bacteria, named IS630-AB1, which was discovered as an IS630 transposon in *Acinetobacter* spp. The phylogenetic relationships of Tc1-OP1, Tc1-MP1 and IS630-AB1 indicated that these transposons had evolved from a common ancestor. Tc1-OP1 is not only the first reported Tc1 transposon in yeast, but also the first reported nonclassical Tc1 transposon. Tc1-OP1 is the largest of IS630/Tc1/mariner transposons reported to date and significantly different from others. Notably, Tc1-OP1 encodes a serine-rich domain and a transposase, extending the current knowledge of Tc1 transposons. Although the intact Tc1-OP1 includes two TIRs, the excision sites and one single ORF, our results did not exclude the possibility that Tc1-OP1 resulted from a fortuitous capture event which had inactivated the transposon. Tc1-OP1 can be used to develop new gene transfer systems, particularly for *Ogataea* spp., as many of them have been genetically engineered for industrial application.

The most important contribution of the present study is the discovery of Tc1-OP1. Both Tc1 transposons in fungi (Tc1-OP1 and Tc1-MP1) are intact, while Tc1 transposons in animals (Tc1-CE1) and bacteria (IS630-AB1) are not. As most of the transposons have been degenerated during evolution and may not be intact after a certain degree of degeneration, the discovery of intact transposons is a rare probability event. Tc1-OP1, Tc1-MP1, Tc1-CE1, and IS630-AB1 have only two, 18, 30 and one full-length copies in the host genomes, respectively. Here, the homologs covered more than 90% of Tc1-OP1, Tc1-MP1, Tc1-CE1, and IS630-AB1 are counted as one full-length copy. Fortunately, two copies of Tc1-OP1 are intact, providing us the very real chance to reveal a putative polyprotein in Tc1/mariner transposons. Tc1-OP1, Tc1-MP1 and IS630-AB1 can be used as reference sequences to facilitate the identification of IS630/Tc1/mariner transposons. More Tc1/mariner transposons will be identified in yeast, following our discovery.

The identity of domainX is still undetermined. There is still the possibility that domainX belongs to the serine or aspartic protease superfamilies, as the percentages of S and D in domainX are 1.96 and 1.12 times higher than those in the Tc1-OP1 transposase, respectively. The serine and aspartic proteases belong to two of the four superfamilies of proteolytic enzymes. In yeast, only one Ca2 + −dependent serine protease has been reported and it belongs to a serine endopeptidase family named the proprotein convertase subtilisin/kexin (PCSK), which is featured by D, H, and S at the active site. The homologs of this serine protease include KEX2 (UniprotKB: P13134) in *S. cerevisiae* and KEX1 (UniprotKB: W1QE83) in *O. parapolymorpha* DL-1. KEX2 and KEX1 cleave protein precursors at C-terminals of the cleavage sites KR, RR or PR. Further analysis revealed that PCC between aa composition of domainX and that of KEX1 reached 0.82. Although a “KR|” (717–718 aa) was detected in the junction region, the CHS active site may not form in the 3D structure of domainX. Aspartic proteases are usually present in retrotransposons and they are featured by two aspartate residues that make up the catalytic machinery. The comparison was performed between domainX with aspartic proteases in yeast, including YPS1 (UniprotKB: P32329) in *S. cerevisiae* and Yapsin 1 (UniprotKB: W1QAF4) in *O. parapolymorpha* DL-1. YPS1 and Yapsin 1 are highly conserved in their aa sequences, however, significantly different from domainX. Two catalytic aspartate residues are located at the 101D and 371D of YPS1 and 84D and 352D of Yapsin 1, respectively, while they are most likely to be 155D and 421D in domainX, which, however, were not close to each other in the 3D structure (Supplementary File S2). In addition, no cleavage sites of aspartic proteases were detected in the junction region between domainX and the transposase. Therefore, domainX is more likely to function in interactions with other proteins or RNAs than function as a serine or aspartic protease.

## Methods and materials

The reference genomes of *O. polymorpha* HU-11/CBS4732 (GenBank: CP073033-40), NCYC495 (GenBank: NW_017264698–704), DL-1 (GenBank: CP080316-22), *C. elegans* (RefSeq: NC_003279–84), *M. piriformis* (GenBank: OW971867-72) and *S. cerevisiae* (Assembly: GCF_000146045.2) were downloaded from the NCBI GenBank or RefSeq database for the analysis in local servers. The reference genome of *C. elegans* was analyzed using the UCSC genome browser and tools. Another genome of *O. polymorpha* DL-1 (RefSeq: NC_027860–66) was used to confirm the results obtained using the DL-1 genome (GenBank: CP080316-22). The nt sequences of Tc1-OP1, Tc1-MP1, Tc1-CE1, and IS630-AB1 are located in the genomes of *O. polymorpha* DL-1 (CP080317: 544314–549,972), *M. piriformis* (OW971871: 2795447–2,793,760), *C. elegans* (NC_003283: 17805799–17,807,409) and *A. baumannii* CAM180-1 (CP044356: 173741–174,626). InterProScan (Jones et al., 2014) v5.56–89.0 was used to predict protein domains against InterPro consortium member databases with default parameter setting. The repeats in all the analyzed genomes were detected using the software RepeatMasker v4.1.2 with the RepBase v20181026 and Dfam v3.6 databases. The software BLAST v2.12.0 was used to search for homologs in a local NCBI NR database with default parameter setting. Signal peptides of protein precursors were detected using SignalP v5.0. The secondary structures of putative proteins were predicted using PSIPRED (Buchan and Jones, 2019) v4.0. The 3D structures of putative proteins were predicted using trRosetta (Yang et al., 2020), trRosettaX, AlphaFold v2.2.0 and RoseTTAFold v1.1.0. The top 5 models of the Tc1-OP1 protein predicted by AlphaFold had low per-residue confidence scores (pLDDTs) around 50 which indicated that most of the protein regions may be unstructured, likewise, those predicted by RoseTTAFold also had low quality scores about 0.3. We had to use trRosetta to improve the prediction of the Tc1-OP1 protein for a better performance. The top 1 model (Supplementary File S2) of the Tc1-OP1 protein (139–1,155 aa) predicted by trRosettaX had the best template modeling (TM) score of 0.334, although it was much lower than the TM scores of Tc1-MP1 and Tc1-CE1 transposases that are 0.62 and 0.688, respectively. DaliLite v5 and 3D-BLAST vbeta102 were used for protein structural alignment and structure database search. The analysis and plotting of protein structures were performed using PyMOL v2.5.26; The neighbor joining (NJ) analyses were performed using MEGA (Kumar et al., 1994) v7.0.26; The maximum likelihood (ML) and Bayesian inference (BI) analyses were performed using PhyloSuite (Zhang et al., 2020) v1.2.2. Statistics and plotting were conducted using the software R v2.15.3 with the Bioconductor packages (Gao et al., 2014). All other data processing were carried out using Perl scripts.

## Data availability statement

The original contributions presented in the study are included in the article/Supplementary material, further inquiries can be directed to the corresponding authors.

## Author contributions

SG conceived the project and drafted the manuscript. SG and YH supervised the present study. GD, TY, and CL performed the programming. JCh, JCu, and HX analyzed the data. WL prepared the figures, tables, and supplementary files. SG and WB revised the manuscript. All authors have read and approved the manuscript.

## Funding

This work was supported by West Light Foundation of the Chinese Academy of Sciences to YH, the Natural Science foundation of China (3181001050) to WB and the Tianjin Applied Basic Research Foundation (21JCYBJC00630) to Ning Zhang. The funding bodies played no role in the design of the study and collection, analysis, and interpretation of data, or in writing the manuscript.

## Conflict of interest

The authors declare that the research was conducted in the absence of any commercial or financial relationships that could be construed as a potential conflict of interest.

## Publisher’s note

All claims expressed in this article are solely those of the authors and do not necessarily represent those of their affiliated organizations, or those of the publisher, the editors and the reviewers. Any product that may be evaluated in this article, or claim that may be made by its manufacturer, is not guaranteed or endorsed by the publisher.
